# Pregnancy outcome at advanced maternal age in a group of African women in two teaching Hospitals in Yaounde, Cameroon

**DOI:** 10.11604/pamj.2013.14.134.2315

**Published:** 2013-04-07

**Authors:** Jean Dupont Kemfang Ngowa, Anny-Nadege Ngassam, Julius Sama Dohbit, Celestine Nzedjom, Jean Marie Kasia

**Affiliations:** 1 Department of Obstetrics and Gynecology, Faculty of Medicine and Biomedical Sciences, University of Yaounde I/Yaounde General Hospital, P.O. Box 5408, Yaounde, Cameroon; 2 Department of Obstetrics and Gynecology, Faculty of Medicine and Biomedical Sciences, University of Yaounde I, P.O. Box 1364, Yaounde, Cameroon; 3 Department of Pediatrics, Faculty of Medicine and Biomedical Sciences, University of Yaounde I, P.O. Box 1364, Yaounde, Cameroon

**Keywords:** Advanced maternal age, pregancy outcome, perinatal outcome

## Abstract

**Introduction:**

Women older than 40 years have been termed “advanced maternal age” and considered to be at risk of adverse pregnancy outcome. This study aimed to examine the obstetrical outcomes among primiparous and multiparous African advanced maternal age women.

**Methods:**

We conducted a retrospective cohort study study at two teaching hospitals at Yaounde, Cameroon. From the hospital records, obstetrical characteristics of 585 consecutive women aged 40 or above who delivered from January 2007 to December 2011 were compared with those of 1816 younger mothers aged 20 to 29 years as control cases. Associations between maternal age and selected obstetrical variables were assessed with the contigency *X*^2^ test or two-tailed Fisher exact test.

**Results:**

Primiparous and multiparous advanced maternal age were more likely to undergo cesarean delivery than were their younger counterparts (38.5% vs 13.5%, RR=2.85, p<0.05 and 16.1% vs 9.1%, RR=1.76, p<0.05). Older primiparous women had similar perinatal outcomes than their younger counterparts. Older multiparous women had increased incidence of preeclampsia/eclampsia (2.4% vs 0.6%, RR=4, p<0.01); antepartum hemorrhage (1.8% vs 0.8%, RR=2.25, p<0.01); fetal distress (3.5% vs 1.3%, RR=2.69, p<0.01); fetal death (3.5% vs 1.6%, RR= 2.18, p<0.05); postpartum hemorrhage (2.4% vs 1.2%; RR=2, p<0.05); preterm delivery (12% vs 9.2%, RR=1.30, p<0.05); low birth weight (11% vs 7.7%, RR=1.42, p<0.05); admission to special care neonatalogy unit(14.1% vs 10.2%, RR=1.38, p<0.05); low Apgar scores at 1min and 5min; and perinatal mortality (3.5% vs 1.6, RR=2.18, p<0.05).

**Conclusion:**

Advanced maternal age women are at higher risk to cesarean delivery. Increased risk of antepartum and intra partum complications among multiparous advanced maternal age women were associated to adverse perinatal outcome. Our results are in concordance with the view that increased risk of adverse perinatal outcome with advanced maternal age is indirectly related to age through the increased risk of obstetrical complications associated with age.

## Introduction

Women older than 35 years have traditionally been termed “advanced maternal age’, although trends in published studies had advanced this age to 40 years [1]. The average age at childbearing in UK has risen sharply over the last decade and 18% of all pregnant women are now aged 35 or above, compared with 8% in 1990. Similar trends are noted in other parts of the world. This is attributed mainly to a confluence of changing social trends such as pursuance of professional careers and delaying of marriages, as well as to the advancement in assisted reproductive technology [1]. Traditionally, these advanced maternal age women are considered to have higher incidence of obstetric complications and adverse pregnancy outcomes than younger pregnant women [1]. Some controversy still exists in the literature on the pregnancy outcomes at advanced maternal age, some researchers [2–4] have suggested compromised pregnancy outcome; others [5–8] have reported comparable outcome for this subgroup.

Delayed childbearing at advanced maternal age may be attributed to several reasons, late marriage, delayed conception due to infertility, academic and career opportunities, desire for large family, ineffective or lack of family planning and longer life expectancy [8, 9]. Contrary to developed countries where advanced maternal age women are more often primiparous, childbearing at advanced maternal age is more common among multiparous women in developing countries as a result of factors such as lack or ineffective family planning methods, favorable cultural disposition towards large family sizes and poverty [8, 10].

The current study was aimed to evaluate the obstetrical outcomes among primiparous and multiparous African women aged 40 or above and compared them with their younger counterparts aged 20-29 years at two teaching hospitals in Cameroon.

## Methods

This was a retrospective cohort study conducted at the Yaounde General Hospital and Yaounde Gynecology, Obstetrics and Pediatrics Hospital, two teaching hospitals at Yaounde, Cameroon, central Africa.

All consecutive women aged 40 or above who delivered from January 2007 to December 2011 in these hospitals were recruited as study group alongside young mothers aged 20 to 29 years as control group. For a delivery from an advanced maternal age woman, the next three deliveries from women aged 20-29 years were selected as control group. Thus, assuming approximately 6.0% exposure risk of perinatal complications (intrauterine death, low birth weight, low Apgar scores and early neonatal death within 7days of life) among mothers aged 40 or above and 2.5% exposure risk among younger mothers aged 20 to 29 years, a sample size of 510 cases for study group and 1,530 cases for control group will have 90% power to detect a 3.5% difference in exposure risk between the two groups at 95% confidence interval. These exposure risks were obtained from the pilot study carried out in the maternity units of these two hospitals and were close to 6.1% and 3.2% risk of intra uterine fetal growth retardation respectively for advanced maternal age women and younger counterparts in one study at the Yaounde University Hospital Center as reported by Tebeu et al. [17]. Because of increased risk of the adverse outcome in multiple pregnancies, only the singleton pregnancies were included.

Data was obtained from available hospital records. We assessed the following obstetrical characteristics and complications; maternal age, gestational age at birth, birthweight, parity, mode of delivery (vaginal, instrumental, cesarean section), malpresentation, preeclampsia/ eclampsia, antepartum hemorrhage, fetal disproportion, fetal distress (defined as persistent or repetitive abnormal fetal heart rate), fetal death (defined as death of fetus at 28weeks of gestation and above or weighing at least 1000g), postpartum hemorrhage (defined as estimated blood loss exceeding 500ml for vaginal birth or 1000ml for cesarean section), preterm delivery (less than 37 weeks and more than 27 weeks), post term pregnancy, perineal tears, episiotomy, low birthweight ( 4000g at term), admission to special care neonatalogy unit, low Apgar score (Apgar score at 1min < 7, Apgar score at 5min

The two groups of maternal age were stratified and analyzed according to parity (primiparous and multiparous). The frequency of antepartum, intrapartum, and postpartum characteristics and complications were assessed in each group. Associations between maternal age and selected obstetrical factors were assessed with the contigency *X* ^2^ test or two-tailed Fisher exact test when the frequencies were small. p<0.05 was considered statistically significant. The researchers obtained permission to carry out this study from the two hospitals management team represented in each hospital by the head of the medical team.

## Results

During the study period, 15,855 women gave birth in the two teaching hospitals, 585 of these women (03%) were ≥ 40 years old at the time of delivery. There were 39 (07%) primiparous and 546 ( 93%) multiparous women of advanced maternal age compared to 643 (34%) primiparous and 1173 (66%) multiparous women of age 20 to 29years old, who were our control group.

Table 1 shows the obstetrical characteristics of women of the two age groups according to their parity. Advanced maternal age women were more likely to undergo cesarean delivery than were their younger counterparts, regardless of parity. The mean birth weight was significantly (p<0.05) higher among multiparous women aged 40 and above than among the younger controls.

**Table 1:** Obstetrical characteristics by maternal age groups and parity

Characteristics	Primiparous				Multiparous			
	20 – 29 y (n=643)	≥40 y (n=39)	RR	p	20 – 29 y (n=1173)	≥40 y (n=546)	RR	P
Maternal age	24.76±2.65	41.28±1.50	-	NS	26.16±2.33	41.53±1.62	-	NS
Mean birth weight (g)	3107.79±53	3161.11±47	-	NS	3219.74±54	3223.81±64	-	<0.05
Mean gestational age at birth (weeks)	38.72±2.26	38.08±3.45	-	NS	38.78±2.07	38.53±2.34	-	<0.05
**Parity**								
0	1931	39			-	-		
1-2	-	-			1925	159		
3-5	-	-			413	299		
>5	-	-			8	88		
**Mode of delivery**								
Vaginal	453 (70.5%)	21 (53.8%)	-	NS	899 (76.6%)	359 (66.5%)	0.86	<0.05
Instrumental	104 16.1%)	3 (7.7%)	-	NS	170 (14.4%)	94 (17.4%)	-	NS
Cesarean section	87 (13.5%)	15 38.5%)	2.85	<0.05	107 (9.1%)	87 (16.1%)	1.76	<0.05

y= year; RR= relative risk; NS= non significant

The distribution of women according to antepartum and intrapartum complications and parity in the two maternal age groups is shown in Table 2. Antepartum hemorrhage was tenfold higher in advanced maternal age primiparous women than their younger counterparts and twofold more frequent among advanced maternal age multiparous women than their younger counterparts. As well, the incidence of preeclampsia/eclampsia, fetal distress, fetal death, postpartum hemorrhage, preterm delivery were significantly increased in advanced maternal age multiparous women as compared to their younger multiparous counterparts. These complications were similar amongst primiparous women of 40years and above and their younger counterparts.

**Table 2:** Antepartum and intrapartum complications by maternal age groups and parity

	Primiparous						Multiparous					
	20–29 y (n=643)		≥40 y (n=39)				20 – 29 y (n=1173)		≥40 y (n=546)			
	n	%	n	%	RR	P	n	%	n	%	RR	P
Malpresentation	26	4.0	0	0	-	NS	48	4.1	26	4.9	-	NS
Preeclampsia/ eclampsia	7	1.0	0	0	-	NS	7	0.6	13	2.4	4	<0.01
Antepartum hemorrhage	3	0.5	2	5.1	10.2	<0.05	10	0.8	10	1.8	2.25	<0.01
Fetal disproportion	11	1.7	0	0	-	NS	4	0.3	0	0	-	NS
Fetal distress	24	3.7	3	7.7	-	NS	16	1.3	19	3.5	2.69	<0.01
Fetal death	14	2.2	1	2.6	-	NS	19	1.6	19	3.5	2.18	<0.05
Postpartum hemorrhage	7	1.1	0	0	-	NS	14	1.2	13	2.4	2	<0.05
Preterm delivery	59	9.1	5	12 .8	-	NS	108	9.2	64	12.0	1.3	<0.05
Post term delivery	20	3.1	1	2.6	-	NS	42	3.5	14	2.6	-	NS
Perineal tears	160	24.8	7	20.0	-	NS	158	13.4	69	13.7	-	NS
Episiotomy	138	21.5	8	22.9	-	NS	43	3.6	11	2.2	-	NS

The incidence of perinatal outcomes among advanced maternal age women and their younger counterparts is shown in Table 3. Statistically, there was no difference in perinatal outcomes between primiparous advanced maternal age women and their younger counterparts. However, the incidence of low birth weight, admission to special care neonatalogy unit, low Apgar scores (at 1 min and 5 min) and perinatal mortality rates were significantly higher in multiparous advanced maternal age women when compared to those of their younger counterparts.

**Table 3:** Perinatal outcome in advanced maternal age women and younger controls

	Primiparous						Multiparous					
	20-29 y (n=643)		≥40 y (n=39)		RR	P	20-29 y (n=1173)		≥40y (n=546)		RR	P
	n	%	n	%			n	%	n	%		
Low birth weight <2500g	59	9.2	3	8.3	-	NS	91	7.7	55	11	1.42	< 0.05
Macrosomia	20	3.1	1	2.8	-	NS	62	5.3	34	6.8	-	NS
Admission to special care neonatalogy unit	98	15.2	6	15.4	-	NS	120	10.2	76	14.1	1.38	< 0.05
**Low Apgar score**												
Apgar at 1min < 7	99	15.4	10	25.6	-	NS	115	9.8	87	16.3	1.66	< 0.05
Apgar at 5min < 7	72	11.1	4	10.5	-	NS	93	7.9	63	12.5	1.58	< 0.05
Perinatal mortality	14	2.2	1	2.6	-	NS	19	1.6	19	3.5	2.18	< 0.05

## Discussion

Our study shows that nulliparous and multiparous African women who are giving birth at 40years and above are at higher risk for cesarean delivery when compared to their younger (20-29years) counterparts. However, primiparous advanced maternal age women have no adverse perinatal outcome, while multiparous advanced maternal age women are at higher risk for adverse perinatal outcome, as compared to their younger (20-29years) counterparts. The increased incidence of cesarean delivery among older women in our study has been reported by several authors [12–19]. One theory often advanced to explain the increased rate of cesarean delivery among older women is that, ageing leads to inefficient uterine action and dystocia in labor [11]. In our setting, for the elderly primiparous mothers, because of low fecundity and often the history of infertility, both patients and obstetricians usually adopt the more active approach by elective cesarean delivery. This approach should explain the high rate of cesarean delivery in primiparous older women in our study (38.5% vs 13.5%; RR= 2.85; p<0.05). However, the increased incidence of antepartum and intrapartum complications (antepartum hemorrage, preeclampsia/eclampsia and fetal distress) among multiparous advanced maternal age women explains the significant increased cesarean delivery rate in this group. These complications are usually the indications of elective or emergency cesarean section.

There is some controversy regarding the perinatal outcome in the older pregnant woman [22]. Perinatal mortality rates in advanced maternal age women are in most cases associated with multiparity, low socio-economic status, preterm birth, intrauterine growth restriction, congenital anomalies and peripartum complications such as asphyxia, birth injuries and infections. Interestingly, there are strong indications to support the view that the entire age-dependent group, had an increase in perinatal mortality rate caused by obstetrical complications resulting from age-dependent confounders such as hypertension and diabetes [21]. Our study is in accordance with this view because the perinatal mortality was significantly higher among multiparous advanced maternal women when compared to their younger counterparts. Also, there were higher incidences of pre eclampsia/eclampsia, antepartum hemorrhage, fetal distress, fetal death, preterm delivery among multiparous advanced maternal age.

In this study, the primiparous older women have statistically similar perinatal outcome with their younger primiparous women and similar antepartum and intrapartum complications except antepartum hemorrhage. This result was similar to other reports [5, 8]. Olusanya et al in a study of 513 advanced maternal age mothers in a tertiary hospital in Nigeria reported no significant adverse perinatal outcome in advanced maternal age women compared with younger mothers [8]. The authors concluded that advanced maternal age was not necessarily associated with adverse perinatal outcomes in settings with good antenatal follow-up and favorable maternal disposition to planned cesarean section. Otherwise, similar antepartum and intrapartum complications noted in primiparous older women and younger counterparts in this study can be explained by the higher rate of cesarean section among primiparous advanced maternal age women (38,5%) than their younger counterparts(13,5%). This can explain the reduction in the occurrence of intrapartum complications in the group of advanced maternal age women. The tendency of primiparous advanced maternal age women to be more assiduous to antenatal care could have also contributed to reduce antepartum complications in that group.

We found that multiparous women of advanced maternal age were at increased risk for preterm birth, low birth weight infants, low Apgar score [6, 16, 19, 20]. The increased incidence of antepartum and intrapartum complications among multiparous advanced maternal age women explained the adverse perinatal outcomes in this group. So, the increased incidence of fetal distress and preterm birth among multiparous mothers of age 40years and above in this study can be explained by the higher rates of low Apgar scores and admission to special care neonatalogy unit.

This discordance in the perinatal outcome between multiparous and primiparous older women when compared to younger counterparts seems to be a strong indication to support the view that, there is increased risk of adverse pregnancy outcome among advanced maternal age mothers in relation to obstetrical complications resulting from age-dependent factors such as hypertension, diabetes, high parity, uterine myomas and a history of infertility [21]. However, retrospective studies such as this one have substantial limitations and carry considerable risk of ascertainment bias.

## Conclusion

Advanced maternal age women are at higher risk to cesarean delivery when compared to their younger counterparts. Primiparous older women had similar obstetrical complications and perinatal outcomes with their younger counterparts meanwhile multiparous advanced women had adverse perinatal outcomes and increased incidence of pre eclampsia/eclampsia, antepartum hemorrhage and fetal distress. Our findings seem to support the view that increased risk of adverse perinatal outcome with advanced maternal age is indirectly related to age through the increased risk of obstetrical complications associated with age. Further prospective studies are needed in the areas of complications and birth outcomes of pregnancy in advanced age women, so that better management protocols may be developed.
